# Insights into the Unique Lung Microbiota Profile of Pulmonary Tuberculosis Patients Using Metagenomic Next-Generation Sequencing

**DOI:** 10.1128/spectrum.01901-21

**Published:** 2022-02-23

**Authors:** Guohui Xiao, Zhao Cai, Qinglong Guo, Taosheng Ye, Yimin Tang, Peikun Guan, Juanjuan Zhang, Min Ou, Xiangdong Fu, Lili Ren, Minfei Yu, Zhaoqin Wang, Lei Liu, Liang Yang, Guoliang Zhang

**Affiliations:** a National Clinical Research Center for Infectious Diseases, Shenzhen Third People’s Hospital, Southern University of Science and Technology, Shenzhen, China; b Guangdong Key Laboratory of Regional Immunity and Diseases, Shenzhen University School of Medicine, Shenzhen, China; c School of Medicine, Southern University of Science and Technology, Shenzhen, China; d Guangzhou Sagene Biotech Co., Ltd., Guangzhou, China; e Institute of Pathogen Biology (IPB), Chinese Academy of Medical Sciences and Peking Union Medical College, Beijing, China; University of California, San Diego

**Keywords:** tuberculosis, *Mycobacterium tuberculosis*, anti-TB treatment, antibiotic resistance genes, lung microbiota, metagenomics

## Abstract

The microbiota plays an important role in human health and disease development. The lung microbiota profile in pulmonary tuberculosis (TB) patients and the effects of anti-TB treatment on the profile need to be determined thoroughly and comprehensively. This study primarily aimed to determine the lung microbiota profile associated with pulmonary TB and characterize the longitudinal changes during anti-TB treatment. A total of 53 participants, comprising 8 healthy individuals, 12 untreated pulmonary TB patients, 15 treated pulmonary TB patients, 11 cured pulmonary TB patients, and 7 lung cancer patients, were recruited in the present study. Bronchioalveolar lavage fluid (BALF) samples were collected from the above participants, and throat swabs were taken from healthy individuals. Microbiomes in the samples were examined using metagenomic next-generation sequencing (mNGS). Differences in microbiota profiles were determined through a comparison of the indicated groups. Our findings indicated that the BALF samples displayed decreased richness and diversity of the microbiota compared to those of the throat swab samples, and these two kinds of samples exhibited obvious separation on principal-coordinate analysis (PCoA) plots. Untreated pulmonary TB patients displayed a unique lung microbiota signature distinct from that of healthy individuals and lung cancer patients. Our data first demonstrated that anti-TB treatment with first-line drugs increases alpha diversity and significantly affects the beta diversity of the lung microbiota, while it also induces antibiotic resistance genes (ARGs).

**IMPORTANCE** Characterization of the lung microbiota could lead to a better understanding of the pathogenesis of pulmonary TB. Here, we applied the metagenomic shotgun sequencing instead of 16S rRNA sequencing method to characterize the lung microbiota using the BALF samples instead of sputum. We found that alterations in the lung microbiota are associated with TB infection and that anti-TB treatment significantly affects the alpha and beta diversity of the lung microbiota in pulmonary TB patients. These findings could help us better understand TB pathogenesis.

## INTRODUCTION

The disaster caused by Mycobacterium tuberculosis infection has been recorded since early human history, highlighting the major challenges for tuberculosis (TB) control and eradication. Approximately one-fourth of the world’s population has been infected with M. tuberculosis, but only 5 to 10% ultimately develop active TB (1), implying that host and environmental factors play important roles in determining the outcomes of M. tuberculosis infection. Current strategies to remedy TB, especially multidrug-resistant TB, depend heavily on long-term medication (2), which may be accompanied by a high risk of severe adverse drug reactions, particularly anti-TB drug-induced liver injury (AT-DILI) (3, 4). Most of the studies indicate that a complex and dynamic interaction between host and M. tuberculosis contributes to TB pathogenesis, so in addition to canonical pathogen-directed strategies, host-directed therapy is a novel and promising approach to anti-TB treatment, especially for drug-resistant TB, and the host microbiota is considered a potential target for improving the clinical outcomes.

The application of culture-independent techniques to investigate the lung microbiota has changed our previous viewpoint that healthy lungs are sterile. Despite the rapid development of human microbiota research, the number of available studies on the lung microbiota in the context of pulmonary TB remains limited. Most of them used sputum as an indicator for the microbiota of the lung and lower respiratory tract (5–11). However, sputum is easily contaminated by microbes residing in the upper respiratory tract during expectoration, leading to an inability to authentically reflect the profiles of the lung microbiota. To date, there have been only three published studies that have examined the microbiota using bronchioalveolar lavage fluid (BALF) samples, which are closer to the real profile of the microbiota in the lungs (12–14). Among the first-line anti-TB drugs, isoniazid (H), pyrazinamide (Z), and ethambutol (E) are considered to specifically target *Mycobacteria*, while rifampin (R) is a broad-spectrum antibiotic. A few studies have examined the impact of anti-TB treatment on the gut microbiota, while the effects of anti-TB treatment on the lung microbiota have not yet been explored (15, 16). Moreover, almost all of these studies on the lung microbiota are based on the 16S rRNA gene amplicon sequencing method, which has obvious limitations, including potential deviations in microbial composition due to amplification bias and the inability to detect most microorganisms at the species and strain levels (17, 18).

In this study, we employed metagenomic next-generation sequencing (mNGS) to first investigate the differences in lung microbiota profiles between the upper and lower respiratory tracts from healthy individuals through comparison of throat swabs and BALF samples. Then, we characterized the lung microbiota profiles of TB patients by comparison of microbiota in BALF samples from untreated patients who did not receive any antibiotics and healthy individuals or lung cancer patients with similar radiological signs. Finally, we attempted to assess the effects of anti-TB treatment on the lung microbiota profiles through comparison of the indicated groups.

## RESULTS

### Participant characteristics, sequencing statistics, and overview of the microbiota in all groups.

**FIG 1:**
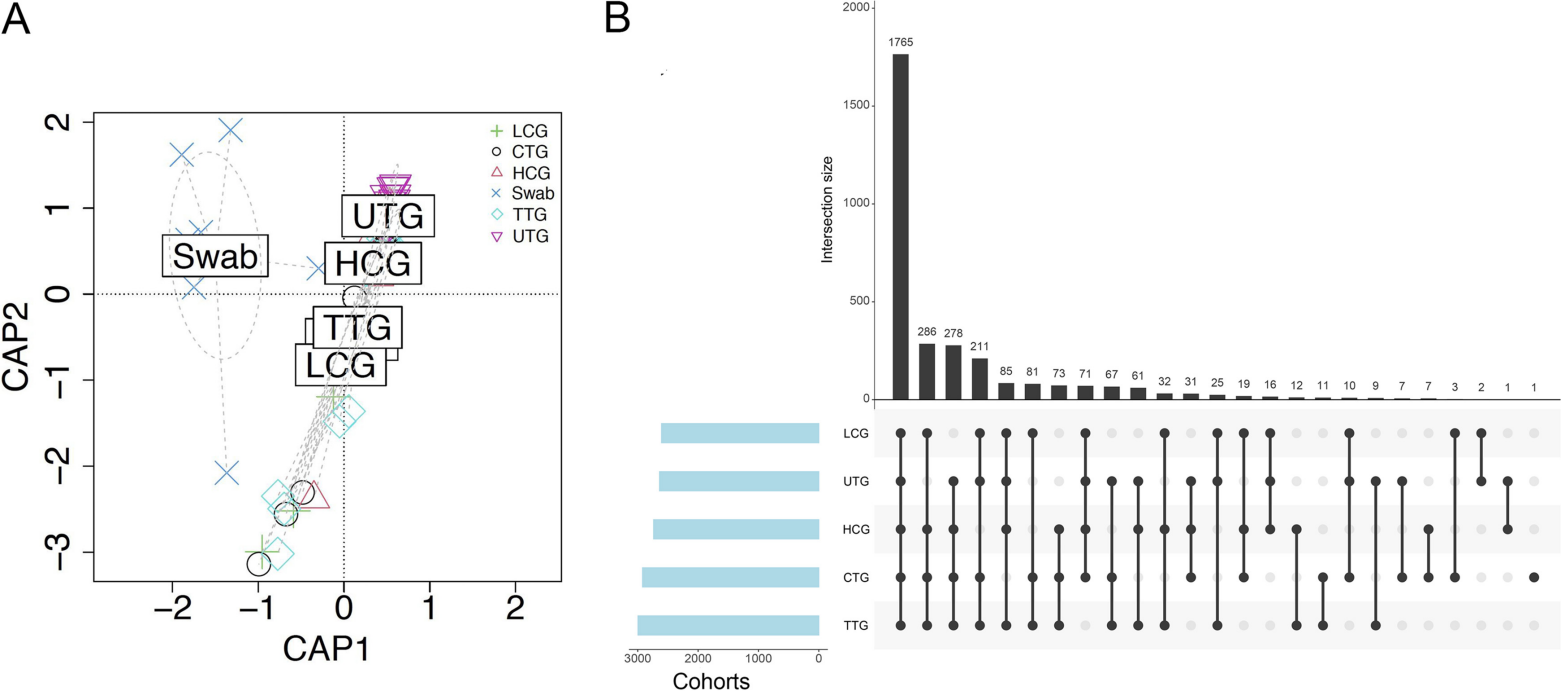
Overview of microbiota within all samples. (A) Constrained analysis of principal coordinates (CAP) using bacterial species relative abundances. The ellipses indicate throat swab samples. (B) UpSet plots were used to determine the common species within the indicated groups. The vertical bar charts represent the number of species contained in each type of group. The vertical bar charts represent the number of species included in each group. The dotted lines show the contained groups.

A total of 53 participants, comprising 8 in the healthy control group (HCG; 3 males, 5 females), 12 in the untreated pulmonary TB group (UTG; 5 males, 7 females), 15 in the treated pulmonary TB group (TTG; 9 males, 6 females), 11 in the cured pulmonary TB group (CTG; 3 males, 8 females), and 7 in the lung cancer patient group (LCG; 5 males, 2 females) were recruited in the present study. The characteristics of the enrolled participants are shown in Table S1. The mean ages of the HCG, UTG, TTG, and CTG were 30.0, 34.6, 37.5, and 29.9 years, respectively. The LCG exhibited the greatest mean age at 58.7. None of the participants in the UTG, the CTG, and the HCG had comorbidities. Two patients in the TTG had diabetes or kidney stones, while three patients in the LCG had either diabetes or hypertension. All participants had no history of smoking. Individuals in the TTG had received anti-TB treatment for 2 weeks to 2.5 months, while individuals in the CTG had received anti-TB treatment for longer than 6 months. All five cohorts exhibited similar average body mass index (BMI). An average of 8.4 × 10^7^ and 2.1 × 10^7^ clean reads were generated per BALF sample and throat swabs, respectively (Table S2). The clean reads were aligned to the reference human genome (GRCh38), and the matching reads were removed. The remaining reads were mapped to bacterial and archaeal databases. The reads for negative control 1 (NC1) and NC2 matched to the bacterial domain were 2.57 × 10^5^ and 7.96 × 10^4^, respectively. The reads for throat swabs from healthy participants matched to the bacterial domain were 5.65 × 10^6^. The reads for BALF samples from the HCG, UTG, TTG, CTG, and LCG matched to the bacterial domain were 1.91 × 10^5^, 2.04 × 10^5^, 3.48 × 10^5^, 4.60 × 10^5^, and 4.67 × 10^5^, respectively (Table S2 and Fig. S1). The raw reads numbers mapped to bacterial species are provided in Table S3. A constrained analysis of principal coordinates (CAP) was performed on all cohort data, and it was found that there was distinct separation of microbiota composition between throat swab and BALF samples at both the genus and species levels (Fig. 1A and Fig. S2A). UpSet plots revealed that 1,765 species and 757 genera were common in BALF samples of all the analyzed groups (Fig. 1B and Fig. S2B).

### Microbiota profiles differ significantly between throat swabs and BALF samples.

**FIG 2:**
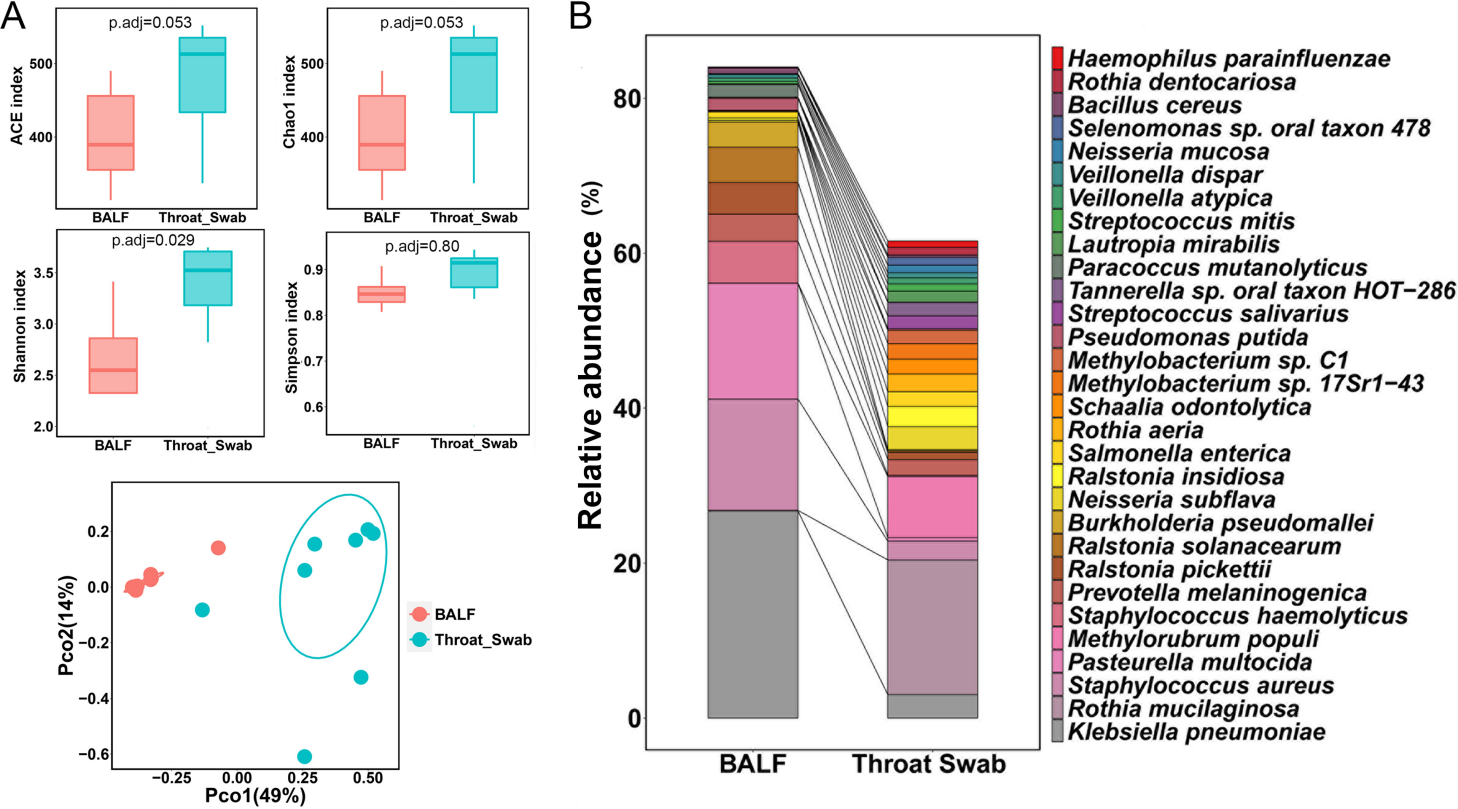
Microbiota profiles differ significantly between throat swabs and BALF samples from healthy controls. (A) Alpha diversity indicated by abundance-based coverage estimator (ACE) index, Chao1 index, Shannon index, and Simpson index at the species level. Adjusted *P* values are listed at the top of the bar charts. Beta diversity depicted by principal-coordinate analysis (PCoA) plot based on Bray-Curtis dissimilarity at the species level. Each dot represents one sample from each group. (B) Bar chart of the top 30 species in the two types of samples. Each color bar represents one species of bacteria.

To investigate differences of microbiota between the upper respiratory tract and lower respiratory tract, we compared the microbiota between throat swabs and BALF samples from healthy participants. The ACE index and Chao1 index in throat swabs were much higher than those in BALF samples at both the species and genus levels (Fig. 2A and Fig. S3). The Shannon index in throat swabs was significantly higher than that in BALF samples at the species level, but there was no significant difference at the genus level (Fig. 2A and Fig. S3), while the Simpson index exhibited no significant differences between these two groups of samples at the species or genus level (Fig. 2A and Fig. S3). Principal-coordinate analysis (PCoA) revealed that the cluster of BALF samples clearly separated from the cluster of throat swabs at both the species and genus levels (Fig. 2A and Fig. S3), suggesting a divergent composition of the microbiota between the upper respiratory tract and lower respiratory tracts.

We further analyzed the top 30 relative abundant species between throat swabs and BALF samples (Fig. 2B and Table S4). The top 30 species in throat swabs and BALF samples accounted for 61.58% and 84.03% of the total bacteria, respectively. Klebsiella pneumoniae, Staphylococcus aureus, and Pasteurella multocida were the three most predominant species in BALF samples, accounting for more than half of the total bacteria, while Rothia mucilaginosa was the most abundant species in throat swabs, accounting for 17.39% of the total bacteria alone.

### The lung microbiota in patients with pulmonary TB differs from that in healthy people and lung cancer patients.

**FIG 3:**
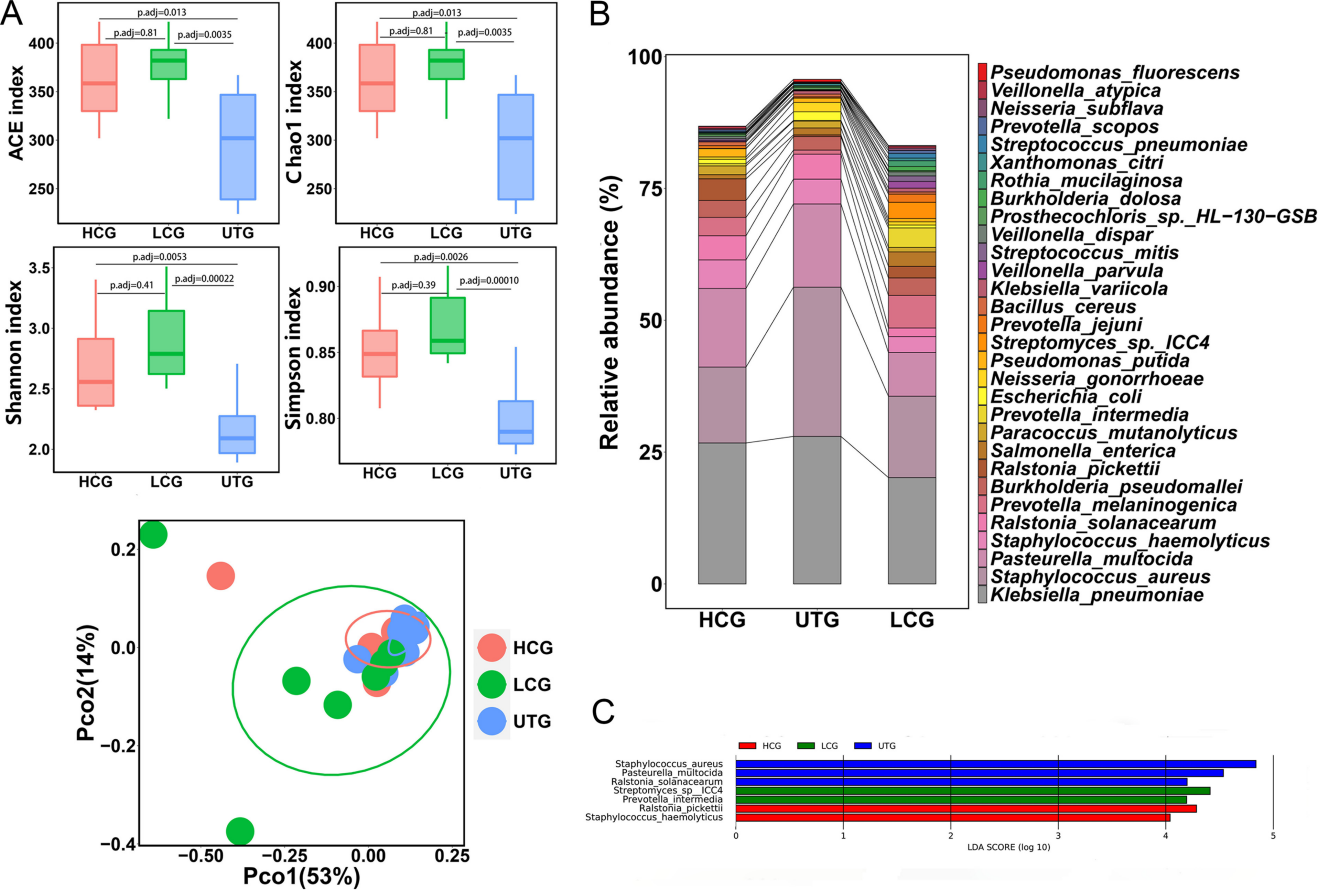
Comparison of lung microbiota among the UTG, HCG, and LCG samples. (A) Alpha diversity indicated by the ACE index, Chao1 index, Shannon index, and Simpson index at the species level. Adjusted *P* values are listed at the top of the bar charts. Beta diversity depicted by PCoA plot based on Bray-Curtis dissimilarity at the species level. Each dot represents one sample from each group. (B) Bar chart of the top 30 species in the HCG, UTG, and LCG samples. Each color bar represents one species of bacteria. (C) Differentially enriched taxa identified by linear discriminant analysis effect size (LEfSe) among the three groups. The length of the column represents the influence of significantly different species on relative abundance (LDA scores [log_10_] > 4). HCG represents the healthy control group; UTG represents the untreated TB group; LCG represents the lung cancer group.

To investigate the association of the lung microbiota with M. tuberculosis infection, we compared the microbiota of the UTG to that of the HCG and the LCG. We observed that the ACE index, Chao1 index, Shannon index, and Simpson index in the UTG were much lower than those in the HCG and the LCG at both the species and genus levels (Fig. 3A and Fig. S4A). In contrast, the four indices exhibited no significant difference between the HCG and the LCG (Fig. 3A and Fig. S4A). We then plotted PCoA to compare the overall structure of the lung microbial composition among these three groups. PCoA illustrated that the UTG samples were hardly separated from the HCG and LCG samples in the plot at the species level but could be distinguished from the other two groups at the genus level (Fig. 3A and Fig. S4A). However, permutational multivariate analysis of variance (PERMANOVA) revealed that the UTG showed significant compositional differences compared to the HCG and the LCG at both the genus and species levels (for species level, UTG versus HCG, PERMANOVA, adjusted *P* value [*P*_adj_] = 0.0033, UTG versus LCG, PERMANOVA, *P*_adj_= 0.0033; for genus level, UTG versus HCG, PERMANOVA, *P*_adj_= 0.01, UTG versus LCG, PERMANOVA, *P*_adj_= 0.01).

We further analyzed the relative abundance of the top 30 most abundant taxa at species levels. The top 30 species in the HCG, the UTG, and the LCG accounted for more than 80% of the total bacteria (Fig. 3B and Table S5). We observed that Klebsiella pneumoniae, Staphylococcus aureus, and Pasteurella multocida were the three most predominant species within the three groups, accounting for 56.04%,72.06%, and 43.89% of the total bacteria, respectively (Fig. 3B). We found that the HCG and LCG were enriched with Prevotella melaninogenica and Ralstonia pickettii, while the UTG was enriched with S. aureus and Neisseria gonorrhoeae. The heatmap based on the top 100 species comprehensively and intuitively displayed the differences in the UTG compared to the HCG and the LCG (Fig. S4B). We next employed linear discriminant analysis using linear discriminant analysis effect size (LeFSe) to identify distinct species which have the potential as biomarkers to diagnosis TB or lung cancer. LEfSe analysis showed that three, two, and two specific species were enriched in the UTG, the LCG, and the HCG, respectively (Fig. 3C), when the linear discriminant analysis (LDA; log_10_) was set above 4.0.

### Anti-TB treatment significantly alters the lung microbiota.

**FIG 4:**
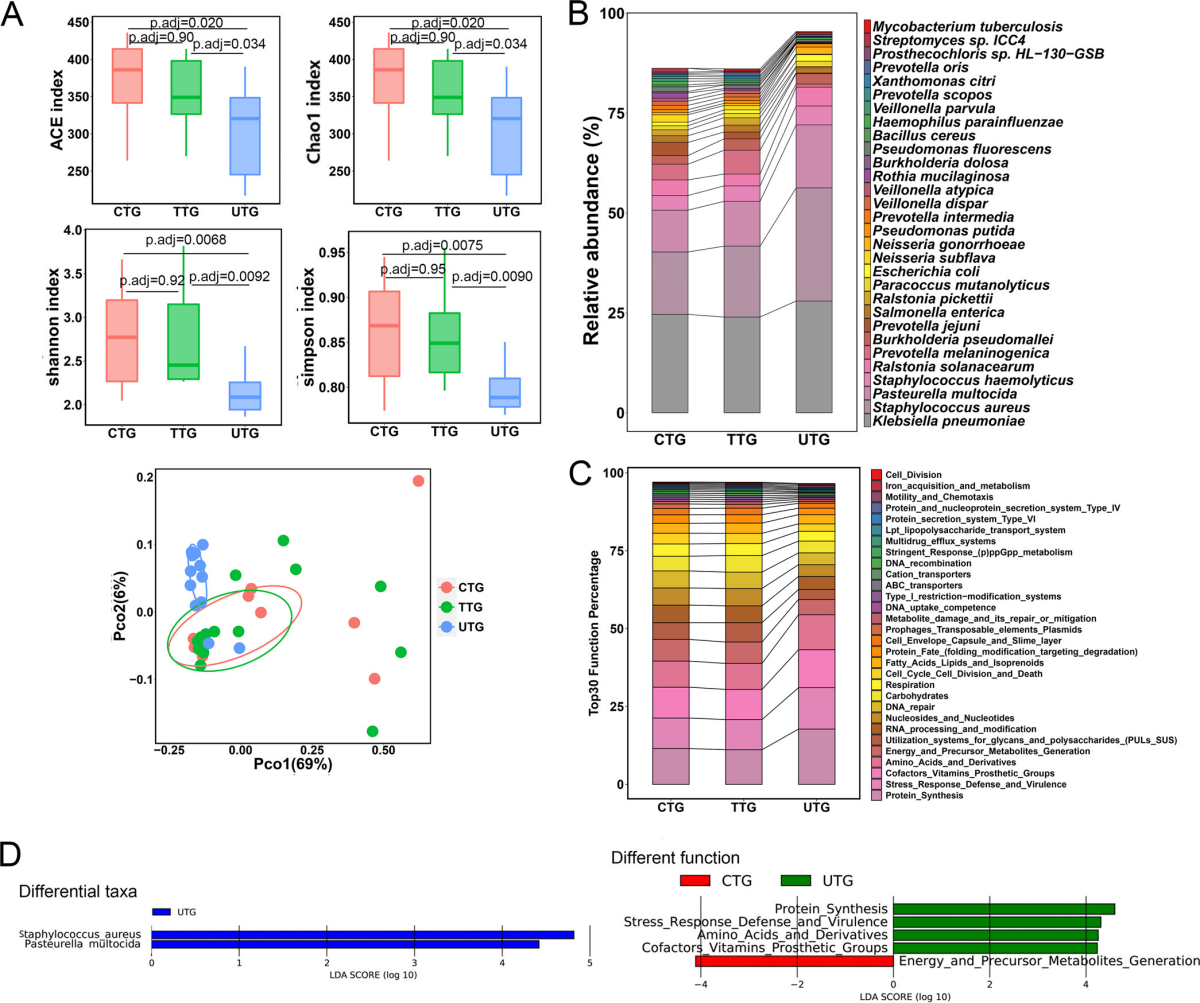
Anti-TB treatment significantly alters the lung microbiota. (A) Alpha diversity indicated by the ACE index, Chao1 index, Shannon index, and Simpson index at the species level. Adjusted *P* values are listed at the top of the bar charts. Beta diversity depicted by PCoA plot based on Bray-Curtis dissimilarity at the species level. Each dot represents one sample from each group. (B) Bar chart of the top 30 species in the UTG, TTG, and CTG. Each color bar represents one species of bacteria. (C) Bar chart of top 30 SEED functions in the UTG, TTG, and CTG. (D) Differentially enriched taxa identified by linear discriminant analysis effect size (LEfSe) among the three groups. The length of the column represents the influence of significantly different species on relative abundance (LDA scores [log_10_] > 4). UTG represents the untreated TB group; TTG represents the treated TB group; CTG represents the cured TB group.

To determine the effects of anti-TB treatment on the lung microbiota, we compared the microbiota in BALF samples from the UTG, TTG, and CTG. We observed that the ACE index, Chao1 index, Shannon index, and Simpson index in the UTG were significantly lower than those in the TTG and the CTG at both the species and genus levels (Fig. 4A and Fig. S5A). No statistical significance in indices was observed between the TTG and the CTG (Fig. 4A and Fig. S5A). PCoA illustrated that the TTG and CTG samples were scattered and extensively overlapped with each other at both the species and genus levels (Fig. 4A and Fig. S5A). PERMANOVA also demonstrated that there were no significant compositional differences in the lung microbiota between the TTG and the CTG samples (PERMANOVA, *P*_adj_= 0.82 at the species level; PERMANOVA, *P*_adj_= 1 at the genus level). The UTG samples were clustered together and positioned away from both the TTG and CTG samples at both the species and genus levels in the PCoA plots (Fig. 4A and Fig. S5A), consistent with the PERMANOVA results (for species level, UTG versus TTG, PERMANOVA, *P*_adj_= 0.015, UTG versus CTG, PERMANOVA, *P*_adj_= 0.0033; for genus level, UTG versus TTG, PERMANOVA, *P*_adj_= 0.11, UTG versus CTG, PERMANOVA, *P*_adj_= 0.04). These results implied the diversity in the microbial profiles among these sample groups, indicating the extensive influence and alteration of anti-TB treatment on lung microbial composition.

The CTG and the TTG exhibited similar compositions of the top 30 most abundant species, but they were different from those of the UTG (Fig. 4B). The top 30 species in the TTG or the CTG accounted for more than 85% of the total bacteria, while these species in the UTG accounted for more than 95% of the total bacteria (Fig. 4B). Among the three groups, K. pneumoniae, S. aureus, and P. multocida were the three most abundant species. The total abundance of the three species accounted for 50.72%, 52.92%, and 72.06% of the total bacteria in the CTG, the TTG, and the UTG, respectively (Fig. 4B and Table S6). We observed that P. melaninogenica, Prevotella jejuni, R. pickettii, Neisseria subflava, and Prevotella intermedia were enriched in the TTG and CTG, while S. aureus, Pasteurella multocida, Escherichia coli, and Neisseria gonorrhoeae were enriched in the UTG (Fig. 4B). The heatmap based on the top 100 species revealed that the overall taxonomic profile in the UTG was distinct from that in the TTG and the CTG (Fig. S5B). The TTG and CTG shared similar patterns of microbial compositions (Fig. S5B).

To identify the specific bacterial species associated with anti-TB treatment, we applied linear discriminant analysis using LEfSe to analyze microbial contents among the three groups. Two specific species were significantly enriched in the UTG, with an LDA (log_10_) of >4. We observed that S. aureus, a potential respiratory pathogen, was strongly enriched in the UTG compared to that in the TTG or the CTG (Fig. 4D).

SEED functional analysis of the metagenome demonstrated that the TTG and the CTG had similar profiles of the top 30 functions, but they were slightly different from those of the UTG (Fig. 4C). LEfSe analysis demonstrated that SEED functions related to protein synthesis, stress response defense and virulence, amino acids and derivatives, and cofactor vitamin prosthetic groups were significantly enriched in the UTG (Fig. 4D). The function related to energy and precursor metabolite generation, which was observed to be enriched in the HCG above, was found to be enriched in the CTG (Fig. 4D).

### Anti-TB treatment increases the diversity and abundance of ARGs in TB patients.

**FIG 5:**
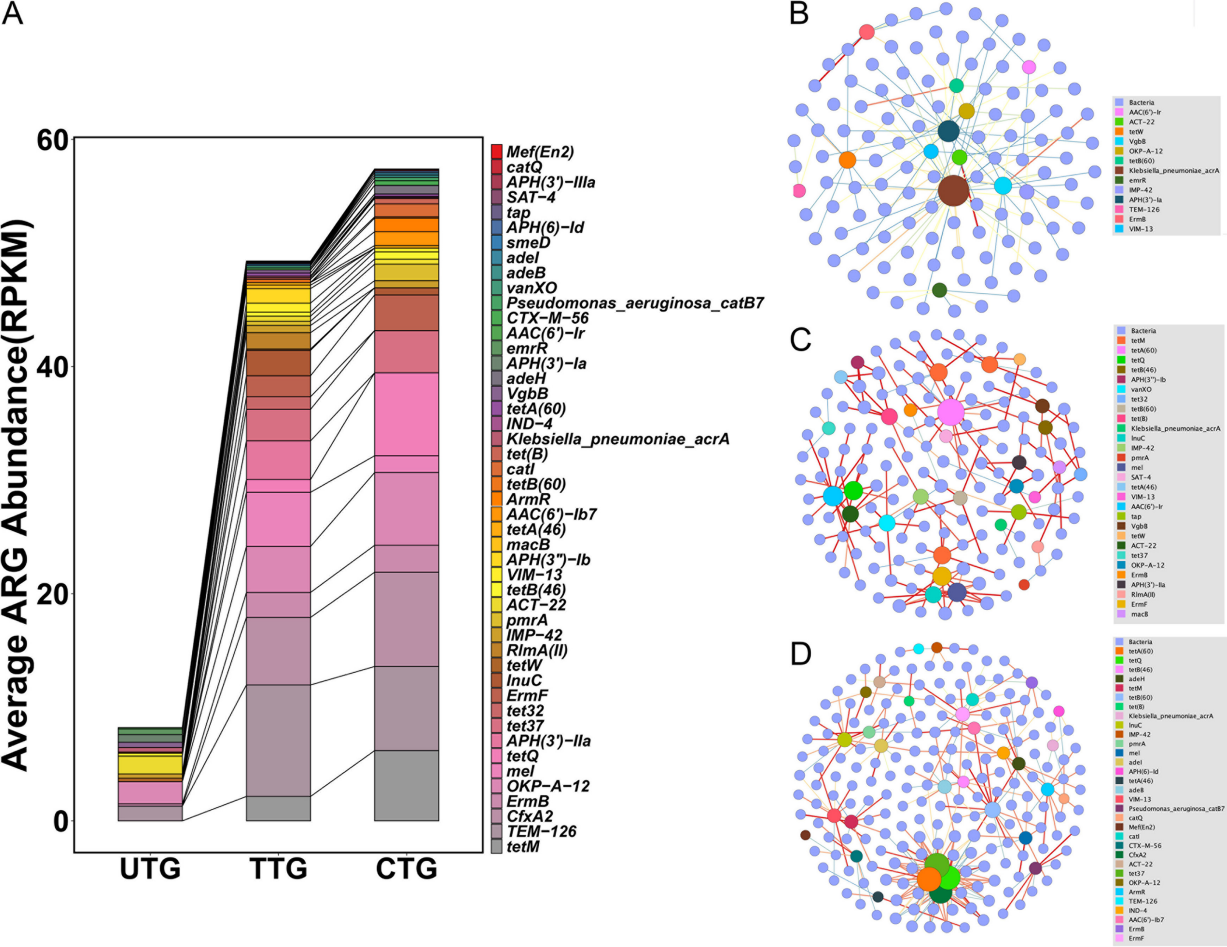
Anti-TB treatment increases the diversity and abundance of ARGs. (A) Bar chart of the abundance of ARGs based on drug classes. (B, C, and D) Cooccurrence network of bacterial species and antimicrobial resistance genes in the UTG (B), TTG (C), and CTG (D) samples based on Spearman correlation index greater than 0.8 in samples. Blue circles, bacteria; colored circles, antimicrobial resistance genes. The blue to red color of edges with increasing thickness indicates the increase in the correlation between two nodes. The UTG undirected network with average degree 1.916, modularity of 0.797, average pathlength of 5.734. The TTG undirected network with average degree 2, modularity of 0.905, average pathlength of 3.535. The CTG undirected network with average degree 3.719, modularity of 0.573, average pathlength of 3.842.

Patients with drug-susceptible tuberculosis were prescribed long-term antibiotic regimens that last at least 6 months as recommended by the WHO. We therefore examined whether long-term antimicrobial exposure in the lung microbiota affects antimicrobial resistance. The average abundance of antibiotic resistance genes (ARGs) per group according to antibiotic class is summarized in Fig. 5A and Table S7. A total of 48 ARGs were detected among these three groups. The lung resistome were dominated by beta-lactam resistance genes (*TEM-126*, *CfxA2*, *OKP-A-12*, *IMP-42*, *ACT-22*, *VIM-13*), followed by tetracycline resistance genes (*tetM*, *tetQ*, *tet32*, *tet37*, *tetW*) and macrolide-lincosamide-streptogramin B (MLSB) resistance genes (*ErmB*, *mel*, *ErmF*). The CTG samples exhibited the highest abundance of ARGs. The TTG had a slightly lower abundance of ARGs than the CTG. The UTG had much less ARG abundance than either the TTG or the CTG. Many resistance genes primarily conferring tetracycline resistance represented in the TTG and the CTG were absent from the UTG. The tremendously increased number of resistance genes in the TTG and CTG was consistent with an increase in medication time, suggesting that long-term anti-TB drug exposure may promote antimicrobial resistance. In addition, we did not observe anti-TB drug resistance genes enriched in the TTG or the CTG samples. The cooccurrence network analysis revealed a gradually more complicated connection between resistance genes and species from the UTG to the CTG, consistent with the increased diversity and abundance of ARGs (Fig. 5B to D).

### Cured TB patients exhibit similar alpha and beta diversity of the lung microbiota but harbor distinct profiles of ARGs compared to those of healthy controls.

**FIG 6:**
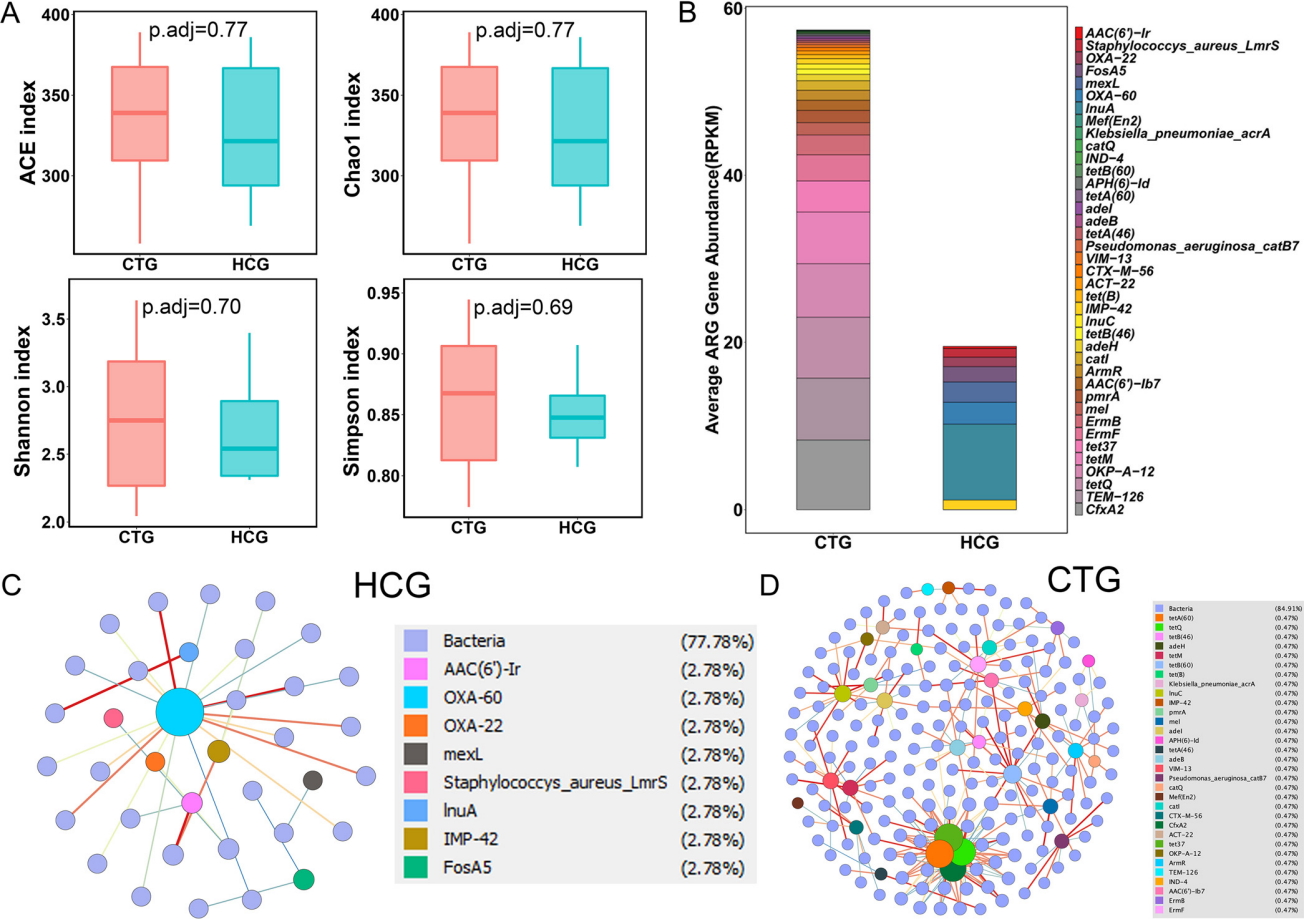
Comparison of the lung microbiota between the CTG and the HCG samples. (A) Alpha diversity indicated by the ACE index, Chao1 index, Shannon index, and Simpson index at the species level. Adjusted *P* values are listed at the top of bar charts. (B) Bar chart of the abundance of ARGs based on drug classes between the two groups. Each bar chart represents one gene. (C and D) Cooccurrence network of bacterial species and ARGs in the HCG and CTG based on Spearman correlation greater than 0.8 in samples. The HCG undirected network with average degree 1.625, modularity of 0.619, average pathlength of 2.618.

The above results indicated that TB infection is associated with reduced alpha diversity while anti-TB treatment increased alpha diversity. To determine how the lung microbiota changes in cured TB patients after long-term anti-TB treatments, we compared the lung microbiota between the HCG and the CTG samples. There were no significant differences in the ACE index, Chao1 index, Shannon index, or Simpson index at species or genus level between the HCG and the CTG groups (Fig. 6A and Fig. S6A). In addition, there were no significant compositional differences between the two cohorts at the genus or species level (PERMANOVA, *P*_adj_= 0.24 at the genus level; PERMANOVA, *P*_adj_= 0.22 at the species level). The HCG and the CTG samples displayed similar compositions of the top 30 most abundant species (Fig. S6B). The top 30 species in both groups accounted for more than 85% of the total bacteria. We did not identify any species that were differentially present between the two groups using LEfSe, even with an LDA of >2.0 (data not shown). These results indicated that cured TB patients displayed structures and compositions of lung microbiota similar to those of healthy people. Interestingly, we found that the CTG displayed much more diversity and abundance of ARGs than the HCG, and the groups shared few common ARGs (Fig. 6B). The cooccurrence networks of bacteria and ARGs in the CTG displayed connections that were much more complicated than those in the HCG (Fig. 6C and D).

## DISCUSSION

Although sputum samples have been used extensively as indicators of lung microbiota to study respiratory diseases, the samples are inevitably contaminated by the pharyngeal microbiota during the collection process (19–22). Some previous studies have demonstrated that pharyngeal contamination has little effect on the microbiota harvested by the bronchoscopy (23–25). We therefore chose to use bronchoalveolar fluids collected by bronchoscopy to investigate the lung microbiota in participants. Studying the lung microbiota in healthy individuals is necessary to determine the associations between microbial changes and respiratory diseases. However, obtaining BALF samples from healthy individuals is challenging because bronchoscopy is an invasive collection method. Thus, very few previous works recruited healthy participants as controls to study the lung microbiota in pulmonary TB (12). Here, we mainly recruited healthy volunteers who had provided health care service in the department of TB for more than 5 years. Although these volunteers had no radiological signs of pulmonary TB, due to the nature of their jobs requiring them to work in close contact with active TB patients, they were willing to accept the examination of bronchoscopy for metagenomic analysis to thoroughly exclude earlier M. tuberculosis infection. Our results also confirmed that no reads of M. tuberculosis were found in these healthy volunteers, which had the great significance of psychologically comforting these high-risk individuals.

The divergence of alpha and beta diversity in the throat swabs and BALF samples from healthy participants revealed significant differences in the microbiota between the upper and lower respiratory tracts, suggesting that the lung contains a self-sustaining microbiota. These results also indicated that the microbiota in BALF samples was not contaminated by pharyngeal microbiota during our collection process.

Our results demonstrated that microbiota profile in BALF samples from TB patients exhibited significantly lower diversity and richness than those of healthy participants, which is contradictory to some previous results from sputum samples (6, 11, 26). These differences are likely to be caused by the type of sample. However, another study from Mexico on microbiota from BALF samples demonstrated that TB patients displayed lower diversity but higher richness of lung microbiota compared to those of healthy control and interstitial pneumonia patients (12). These controversial findings could be explained by some factors, such as geographical factor, DNA sequencing methods, and sample size. PERMANOVA revealed that the pulmonary TB patients displayed significant compositional differences compared to healthy controls or lung cancer patients. These results indicated that pulmonary TB patients were associated with a unique lung microbiota profile.

Due to the application of metagenomic sequencing methods, we were able to identify the changed taxon at species level. Our study revealed a high relative abundance of species S. aureus significantly enriched in TB patients. S. aureus is a very common pathogen that can infect multiple organs and cause critical diseases, such as pneumonia, endocarditis, and bacteremia (27, 28). These increased pathogenic bacteria may cause secondary infection in TB patients, which might exacerbate the lung disease. Our findings displayed that the relative abundances of Ralstonia pickettii and P. melaninogenica were significantly lower in the untreated TB patients than in the healthy controls or lung cancer patients. It was reported that R. pickettii is able to catalyze ethionamide into active form against mycobacteria (29). The decreased abundance of *R. pickettii* in TB patients may not be conducive to outcomes of anti-TB treatment. It has been reported that *Prevotella* is associated with better lung function and less inflammation (30, 31). The lower relative abundance of *P. melaninogenica* in the untreated TB patients may weaken lung function and accelerate inflammation. Although the differences in the lung microbiota between healthy controls and pulmonary TB patients were observed, we were unable to determine whether the identified changes were a cause or consequence of M. tuberculosis infection due to the limited evidence in the present study. Further study will be required to investigate the exact relationships between the altered microbiota and M. tuberculosis infection.

Lung cancer and pulmonary tuberculosis are common diseases. Lung cancer and pulmonary TB have similar clinical symptoms and radiological signs in their onset; in particular, there were high proportions of pulmonary TB patients with negative bacteriology, so it is difficult to properly distinguish them. In addition to radiographic evaluation, there have been numerous biomarkers to assist the diagnosis of lung cancer, such as carcinoembryonic antigen (CEA), cytokeratin 19 fragments (CYFRA 21-1), 2-phospho-d-glycerate hydrolase (NSE), squamous cell carcinoma antigen (SCC-Ag), and pro-gastrin-releasing peptide (Pro-GRP) (32–34). Similarly, these differential taxa could be used as potential markers to assist in the diagnosis of TB or lung cancer. However, these species need further confirmation by other independent cohorts before being used as biomarkers.

Antibiotics have significant impacts on the human microbiota, causing a rapid decrease in diversity, especially after broad-spectrum antibiotic treatment (15, 35–37). It has been reported that conventional anti-TB treatment induced significantly decreased diversity and richness of gut microbiota (38). The lung microbiota was supposed to be decreased in diversity and richness, since the anti-TB drug rifampin is a broad-spectrum antibiotic. However, our findings revealed that TB patients taking conventional anti-TB therapy exhibit significantly higher alpha diversity and significantly different beta diversity compared to those of untreated TB patients. These findings are consistent with a recent study performed by Hu and colleagues, which demonstrated that cured TB patients had a higher alpha diversity and had a different community composition of lung microbiota after negative conversion (13). These findings suggested that anti-TB treatment may cause differential effects on the lung and gut microbiota. We observed that the relative abundances of P. melaninogenica, P. jejuni, and P. intermedia were increased after anti-TB treatment. Hu’s studies reported increased *Prevotella* after negative conversion (13, 14). However, they did not identify these specifically changed species from *Prevotella* genus. We also found significantly decreased abundances of S. aureus and P. multocida in response to anti-TB treatment. We speculated that M. tuberculosis infection alters lung microenvironment, which may restrain certain microbes but induce growth for some other bacteria. The increased abundance and diversity of the lung microbiota in treated TB patients and cured patients may be due to the clearance of M. tuberculosis by anti-TB treatment. In addition, we observed that untreated TB patients clustered more closely together on PCoA plots than other groups, suggesting that TB contributes to stabilizing the microbiota structure. Functional metagenomics analysis revealed that anti-TB treatment resulted in strongly decreased relative abundance of virulence-related genes. However, the undefined relationships between these altered lung microbiota caused by anti-TB treatment and the prognosis of tuberculosis require further investigation.

It has been reported that antibiotic treatment reduces gut microbiome diversity but accelerates enrichment of antibiotic resistance genes and different antibiotics have different effects on gut resistome (39, 40). Pulmonary TB treatment often lasts for at least 6 months. Such long-term antibiotic exposure may have a large impact on lung resistomes. Our findings revealed a tremendously increased abundance of ARGs after anti-TB treatment. The increased abundance of ARGs is very likely to be due to the changed microbiota, since the CTG and TTG showed an alpha diversity of lung microbiota significantly higher than that of the UTG. Lung resistomes in TB patients were dominated by beta-lactam resistance genes and with high prevalence of tetracycline resistance genes and MLSB resistance genes, which is not consistent with Micheal Aogain’s report that airway resistome in sputum from healthy people or patients with chronic respiratory disease was dominated by macrolide resistance genes (41). This difference may be due to the collected analyzed sample type. The cured TB patients and treated TB patients displayed different profiles of ARGs. The CTG showed a higher abundance of ARGs than the TTG. The CTG has a higher percentage of tetracycline resistance genes but a lower percentage of MLSB resistance genes, suggesting that different antibiotics exposures have different effects on profile of ARGs in lung of TB patients. In addition, our findings demonstrated that the cured TB patients exhibited alpha and beta diversity similar to those of healthy controls. However, we found that the cured TB patients exhibited an abundance of ARGs higher than that of the healthy people, suggesting that lung microbiota in cured TB patients did not completely return to normal status. This is an important warning worthy of clinical consideration. Although progress has been made in the use of antibiotics to improve the clinical outcomes of pulmonary TB patients, we must be aware of the potential long-term impact of antimicrobial resistance and strictly control drug overuse for the treatment of TB.

There are several limitations in this study that should be noted. First, the number of individuals included was relatively small. Further studies including a larger number of samples are required to confirm our findings. In addition, in this study, we focused only on bacteria and did not analyze viruses or fungi, which are also important for shaping the profile of the lung microbiota and should be considered in future studies.

### Conclusions.

We profiled the differences in microbiota between the upper respiratory tract and lower respiratory tract by comparing the microbiota in throat swabs and BALF samples. Our findings demonstrated that pulmonary TB patients display a unique lung microbiota signature distinct from that of healthy individuals and lung cancer patients. This study exhibited that anti-TB treatment increases alpha diversity and affects beta diversity in the lung microbiota. Our data also indicated that anti-TB treatment accelerates the enrichment of ARGs.

## MATERIALS AND METHODS

### Study design and sample collection.

Five different cohorts of participants were recruited according to the following criteria: (i) the untreated pulmonary TB group (UTG) included patients not taking any antibiotics, (ii) the treated pulmonary TB group (TTG) included patients receiving more than 2 weeks of standard anti-TB treatment, (iii) the cured pulmonary TB group (CTG) included patients with bacterial negative conversion who had finished long-term anti-TB treatment, (iv) the healthy control group (HCG) were health care providers in the TB department who exhibited negative chest radiological signs and interferon gamma (IFN-γ) release assay (IGRA), and (v) the lung cancer patient group (LCG) included patients with diagnoses confirmed by cellular or tissue pathology. All the TTG and CTG participants were sensitive to first-line anti-TB drugs and received the standard regimen of 2 months of HRZE (isoniazid, rifampin, pyrazinamide, and ethambutol) and 4 months of HR (isoniazid and rifampin). Pulmonary TB patients were diagnosed based on clinical symptoms and radiological signs as described previously (42, 43) and had a positive detection for M. tuberculosis in BALF samples indicated by culture, PCR, or GeneXpert approach. All participants were free of HIV and had no history of smoking. Detailed information on each participant is shown in Table S1. BALF samples were collected from all participants by instilling prewarmed sterile saline (20 mL each time, 2 to 6 times) and aspirating (10 to 20 mL each time). To identify the potential contamination from the environment or reagents used for DNA extraction, we collected saline control samples after injection through the bronchoscope using a sterile syringe. Because the amount of DNA extracted from blank is extremely small, which is not enough for construction of sequencing library, the collected saline control samples were then mixed with THP-1 cells for DNA extraction and designated negative control 1 (NC1). Prepared genomic DNA extracted from HeLa cells was used as negative control 2 (NC2) to determine possible contamination during sequencing library construction. These control samples were processed in parallel with the actual samples. Throat swabs were also collected from healthy participants to investigate differences in the microbiota between the upper and lower respiratory tracts. The collected samples were stored at −80°C for microbiota profiling.

### DNA extraction.

Total DNA was extracted from BALF samples using an Omega Biotech Mag-Bind Universal Pathogen 96 kit (Omega Bio-Tek, Inc., Norcross, GA, USA) according to the manufacturer’s protocols. The concentration and quality of extracted DNA were determined using a Qubit dsDNA assay kit (Life Technologies, USA). All extracted DNA was stored at −80°C until further processing.

### Metagenome sequencing.

The above extracted DNA samples were used for metagenome sequencing by Guangzhou Sagene Biotech Co., Ltd. (Guangzhou, China). One microgram of total DNA per sample was used as an input material for sequencing library construction using a NexteraXT DNA sample preparation kit (Illumina, CA, USA). Prepared genomic DNA from HeLa cells was used as negative control 2 (NC2) to identify the possible contamination during sequencing library construction. After quality assessment, the library preparations were sequenced on an Illumina NovaSeq 6000 platform, and paired-end raw reads were generated with a read length of 150 bp.

### Data processing and sequence analysis.

**Preprocessing of raw data.** Raw reads were filtered using fastp 0.19.5 (44) (–detect_adapter_for_pe -W 4 -q 15 -u 40) with other parameters keeping as default settings to remove adaptors and low-quality reads. The quality of processed reads was assessed using fastqc 0.11.5 with default settings (45). The preprocessed reads were mapped to the reference human genome (GRCh38) using bowtie2 with default settings (-un-conc option) (46), and matching reads were removed. Summary of metagenomic sequencing data was provided in Table S2.

**Taxonomic classification.** Taxonomy of the remaining reads was classified using kraken2 (–paired) with kraken2 bacterial and archaeal databases and default settings (47, 48). Taxonomic abundance was normalized using bracken (-r 150 -l G) based on kraken2 output (49).

**Decontamination and filtering of classified data.** Normalized data obtained from bracken during taxonomic classification of each cohort were combined according to study design using the excel vlookup function and were manually checked to ensure that all data were correctly matched to respective taxa. Combined normalized data were filtered and decontaminated using the decontam 1.6.0 R package using prevalence threshold of 0.5 (50) and keeping species that appeared in more than 5 samples. Abundance data were further filtered to remove low-abundance species using a summed abundance of 0.0005 as the threshold for all samples in each comparison for downstream analyses.

**Statistical analysis and visualization of data.** The top 30 species or genera of each comparison were selected and visualized as bar charts using reshap2 1.4.4, tidyverse 1.3.0, dplyr 1.0.0, and the ggplot2 3.3.0 R packages.

Alpha diversity in a group was calculated based on filtered and normalized counts with rarefying to the lowest taxonomy using the vegan 2.5.6 R package. Intergroup differences and significance were calculated using Tukey’s honestly significant difference (HSD) test with a confidence level of 95% and the least significant difference (LSD) test with a *P*_adj_ of <0.05 and were visualized as box plots using the reshape2 1.4.4, ggplot2 3.3.0, devtools 2.3.2, bindrcpp 0.2.2, ggthemes 4.2.4, agricolae 1.3.3, dplyr 1.0.0, and ggpubr 3.3.0 R packages. Principal-coordinate analysis among sample groups was calculated using Bray-Curtis dissimilarity and visualized using vegan 2.5.6, labdsv 2.0.1, ggplot2 3.3.0, and scales 1.1.0 R packages (51–53). Heatmaps of sample groups were generated using the pheatmap package 1.0.12 in R based on the top 100 species. Functional profiles were analyzed using megan6 community edition with SEED mapping file and LCA (lowest common ancestor) filtering parameters of 100 as minimal bit-score and 25 as minimal support (54). Taxonomical biomarkers of each sample group were analyzed using the LEfSe package 1.0 (format_input.py -c 1 -u 2 -o 1000000; run_lefse.py -l 4; plot_res.py –dpi 600 –format pdf) with an LDA score of 4 and a *P* value of <0.05 (55). UpSet plots were generated using the upsetR package 1.4.0 after converting the data frame of average counts of each cohort to a data frame containing only 0/1 values and ordering by frequency of intersection size. PERMANOVA of the cohorts was performed using the adonis function of the vegan package in R software with permutations of 999 and Bray-Curtis dissimilarity. Pairwise analysis between cohorts was performed using the pairwise.adonis function of the vegan package using Bray-Curtis dissimilarity method with the Bonferroni correction as the p.adjusted method. Constrained analysis of principal coordinates (CAP) was performed using the capsale function of the vegan package in R software with Bray-Curtis as the distance method.

**Resistomics analysis.** Resistomes were identified and quantified using shortbred 0.9.5 (56, 57). Specific resistomic markers were generated using the shortbred_identify function (–clustid 0.95 –qclustid 0.95 –markerlength 10) based on the protein homolog model of the CARD database with the uniref90 database as a reference (58, 59). Quantification of antibiotic resistance genes was performed using the shortbred_quantify option (-id 0.80) using resistomic markers generated and normalized to reads per kilobase per million (RPKM) (56). The cooccurrence network analysis was done using Hmisc and igraph R packages. The cooccurrence of bacteria and antibiotic resistance genes was calculated using Spearman correlation index of >0.8 and *P* value of <0.01 using the hmisc package and was visualized using the igraph package in R (60). The generated cooccurrence plots were visualized using gephi 0.9.2 with fruchterman reingold layout and default settings (61). The statistics (average degree, modularity, average pathlength, etc.) of the cooccurrence plots were generated by gephi 0.9.2 during running the plots.

### Ethics approval.

This study was approved by both the Shenzhen Third People’s Hospital Ethical Committee and the Ethics Committee of Southern University of Science and Technology (20200089). Written informed consent was obtained from all participants, and all methods in this study were performed in accordance with the relevant guidelines and regulations.

### Data availability.

All sequence data filtering out the human genome from this study have been uploaded to the National Center for Biotechnology Information sequence read archives under project accession number PRJNA655567.

## References

[1] Emilia V, Fine PEM. 2000. Lifetime risks, incubation period, and serial interval of tuberculosis. Am J Epidemiol 152:247–263. doi:10.1093/aje/152.3.247. 10933272

[2] Michelle H, Robert B. 2018. Updates in the treatment of active and latent tuberculosis. Semin Respir Crit Care Med 39:297–309. doi:10.1055/s-0038-1660863. 30071545

[3] Prasad R, Singh A, Gupta N. 2019. Adverse drug reactions in tuberculosis and management. Indian J Tuberc 66:520–532. doi:10.1016/j.ijtb.2019.11.005. 31813444

[4] Tostmann A, Boeree MJ, Aarnoutse RE, Lange WCMD, Ven A, Dekhuijzen R. 2008. Antituberculosis drug-induced hepatotoxicity: concise up-to-date review. J Gastroenterol Hepatol 23:192–202. doi:10.1111/j.1440-1746.2007.05207.x. 17995946

[5] Zelin C, Yuhua Z, Hong L, Yan Z, Shulin Z, Shenjie T, Xiaokui G. 2012. Complex sputum microbial composition in patients with pulmonary tuberculosis. BMC Microbiol 12:276. doi:10.1186/1471-2180-12-276. 23176186 PMC3541192

[6] Wu J, Liu W, He L, Huang F, Chen J, Cui P, Shen Y, Zhao J, Wang W, Zhang Y, Zhu M, Zhang W, Zhang Y. 2013. Sputum microbiota associated with new, recurrent and treatment failure tuberculosis. PLoS One 8:e83445. doi:10.1371/journal.pone.0083445. 24349510 PMC3862690

[7] Krishna P, Jain A, Bisen PS. 2016. Microbiome diversity in the sputum of patients with pulmonary tuberculosis. Eur J Clin Microbiol Infect Dis 35:1205–1210. doi:10.1007/s10096-016-2654-4. 27142586

[8] Botero LE, Delgado-Serrano L, Cepeda ML, Bustos JR, Anzola JM, Del Portillo P, Robledo J, Zambrano MM. 2014. Respiratory tract clinical sample selection for microbiota analysis in patients with pulmonary tuberculosis. Microbiome 2:29. doi:10.1186/2049-2618-2-29. 25225609 PMC4164332

[9] Sala C, Benjak A, Goletti D, Banu S, Mazza-Stadler J, Jaton K, Busso P, Remm S, Leleu M, Rougemont J, Palmieri F, Cuzzi G, Butera O, Vanini V, Kabir S, Rahman SMM, Nicod L, Cole ST. 2020. Multicenter analysis of sputum microbiota in tuberculosis patients. PLoS One 15:e0240250. doi:10.1371/journal.pone.0240250. 33044973 PMC7549818

[10] Lin D, Wang X, Li Y, Wang W, Li Y, Yu X, Lin B, Chen Y, Lei C, Zhang X, Zhang X, Huang J, Lin B, Yang W, Zhou J, Zeng J, Liu X. 2021. Sputum microbiota as a potential diagnostic marker for multidrug-resistant tuberculosis. Int J Med Sci 18:1935–1945. doi:10.7150/ijms.53492. 33850462 PMC8040397

[11] Cheung MK, Lam WY, Fung WYW, Law PTW, Au CH, Nong W, Kam KM, Kwan HS, Tsui SKW. 2013. Sputum microbiota in tuberculosis as revealed by 16S rRNA pyrosequencing. PLoS One 8:e54574. doi:10.1371/journal.pone.0054574. 23365674 PMC3554703

[12] Vázquez-Pérez JA, Carrillo CO, Iñiguez-García MA, Romero-Espinoza I, Márquez-García JE, Falcón LI, Torres M, Herrera MT. 2020. Alveolar microbiota profile in patients with human pulmonary tuberculosis and interstitial pneumonia. Microb Pathog 139:103851. doi:10.1016/j.micpath.2019.103851. 31715320

[13] Hu Y, Kang Y, Liu X, Cheng M, Dong J, Sun L, Zhu Y, Ren X, Yang Q, Chen X, Jin Q, Yang F. 2020. Distinct lung microbial community states in patients with pulmonary tuberculosis. Sci China Life Sci 63:1522–1533. doi:10.1007/s11427-019-1614-0. 32303963

[14] Yongfeng H, Min C, Bo L, Jie D, Lilian S, Jian Y, Fan Y, Xinchun C, Qi J. 2020. Metagenomic analysis of the lung microbiome in pulmonary tuberculosis - a pilot study. Emerg Microbes Infect 9:1444–1452. doi:10.1080/22221751.2020.1783188. 32552447 PMC7473061

[15] Wang J, Xiong K, Zhao S, Zhang C, Zhang J, Xu L, Ma A. 2020. Long-term effects of multi-drug-resistant tuberculosis treatment on gut microbiota and its health consequences. Front Microbiol 11:53. doi:10.3389/fmicb.2020.00053. 32082283 PMC7002438

[16] Wipperman MF, Fitzgerald DW, Juste MAJ, Taur Y, Namasivayam S, Sher A, Bean JM, Bucci V, Glickman MS. 2017. Antibiotic treatment for Tuberculosis induces a profound dysbiosis of the microbiome that persists long after therapy is completed. Sci Rep 7:10767. doi:10.1038/s41598-017-10346-6. 28883399 PMC5589918

[17] Konstantinidis KT, Tiedje JM. 2007. Prokaryotic taxonomy and phylogeny in the genomic era: advancements and challenges ahead. Curr Opin Microbiol 10:504–509. doi:10.1016/j.mib.2007.08.006. 17923431

[18] Pinto AJ, Raskin L. 2012. PCR biases distort bacterial and archaeal community structure in pyrosequencing datasets. PLoS One 7:e43093. doi:10.1371/journal.pone.0043093. 22905208 PMC3419673

[19] Cox MJ, Allgaier M, Taylor B, Baek MS, Huang YJ, Daly RA, Karaoz U, Andersen GL, Brown R, Fujimura KE, Wu B, Tran D, Koff J, Kleinhenz ME, Nielson D, Brodie EL, Lynch SV. 2010. Airway microbiota and pathogen abundance in age-stratified cystic fibrosis patients. PLoS One 5:e11044. doi:10.1371/journal.pone.0011044. 20585638 PMC2890402

[20] Molyneaux PL, Mallia P, Cox MJ, Footitt J, Willis-Owen SAG, Homola D, Trujillo-Torralbo M-B, Elkin S, Kon OM, Cookson WOC, Moffatt MF, Johnston SL. 2013. Outgrowth of the bacterial airway microbiome after rhinovirus exacerbation of chronic obstructive pulmonary disease. Am J Respir Crit Care Med 188:1224–1231. doi:10.1164/rccm.201302-0341OC. 23992479 PMC3863728

[21] Zhao J, Schloss PD, Kalikin LM, Carmody LA, Foster BK, Petrosino JF, Cavalcoli JD, VanDevanter DR, Murray S, Li JZ, Young VB, LiPuma JJ. 2012. Decade-long bacterial community dynamics in cystic fibrosis airways. Proc Natl Acad Sci U S A 109:5809–5814. doi:10.1073/pnas.1120577109. 22451929 PMC3326496

[22] Rogers GB, Van Der Gast CJ, Cuthbertson L, Thomson SK, Bruce KD, Martin ML, Serisier DJ. 2013. Clinical measures of disease in adult non-CF bronchiectasis correlate with airway microbiota composition. Thorax 68:731–737. doi:10.1136/thoraxjnl-2012-203105. 23564400

[23] Bassis CM, Erb-Downward JR, Dickson RP, Freeman CM, Schmidt TM, Young VB, Beck JM, Curtis JL, Huffnagle GB. 2015. Analysis of the upper respiratory tract microbiotas as the source of the lung and gastric microbiotas in healthy individuals. mBio 6:e00037. doi:10.1128/mBio.00037-15. 25736890 PMC4358017

[24] Dickson RP, Martinez FJ, Huffnagle GB. 2014. The role of the microbiome in exacerbations of chronic lung diseases. Lancet 384:691–702. doi:10.1016/S0140-6736(14)61136-3. 25152271 PMC4166502

[25] Dickson RP, Erb-Downward JR, Martinez FJ, Huffnagle GB. 2016. The microbiome and the respiratory tract. Annu Rev Physiol 78:481–504. doi:10.1146/annurev-physiol-021115-105238. 26527186 PMC4751994

[26] Hong B-y, Paulson JN, Stine OC, Weinstock GM, Cervantes JL. 2018. Meta-analysis of the lung microbiota in pulmonary tuberculosis. Tuberculosis (Edinb) 109:102–108. doi:10.1016/j.tube.2018.02.006. 29559113

[27] Coates T, Bax R, Coates A. 2009. Nasal decolonization of Staphylococcus aureus with mupirocin: strengths, weaknesses and future prospects. J Antimicrob Chemother 64:9–15. doi:10.1093/jac/dkp159. 19451132 PMC2692503

[28] Thomer L, Schneewind O, Missiakas D. 2016. Pathogenesis of Staphylococcus aureus bloodstream infections. Annu Rev Pathol 11:343–364. doi:10.1146/annurev-pathol-012615-044351. 26925499 PMC5068359

[29] Dodge AG, Richman JE, Johnson G, Wackett LP. 2006. Metabolism of thioamides by Ralstonia pickettii TA. Appl Environ Microbiol 72:7468–7476. doi:10.1128/AEM.01421-06. 16997975 PMC1694237

[30] O’Neill K, Bradley JM, Johnston E, McGrath S, McIlreavey L, Rowan S, Reid A, Bradbury I, Einarsson G, Elborn JS, Tunney MM. 2015. Reduced bacterial colony count of anaerobic bacteria is associated with a worsening in lung clearance index and inflammation in cystic fibrosis. PLoS One 10:e0126980. doi:10.1371/journal.pone.0126980. 25992575 PMC4439045

[31] Carmody LA, Caverly LJ, Foster BK, Rogers MAM, Kalikin LM, Simon RH, VanDevanter DR, LiPuma JJ. 2018. Fluctuations in airway bacterial communities associated with clinical states and disease stages in cystic fibrosis. PLoS One 13:e0194060. doi:10.1371/journal.pone.0194060. 29522532 PMC5844593

[32] Du C, Xue W, Dou F, Peng Y, Yao Y, Zhao J, Gu J. 2017. Use of a combination of CEA and tumor budding to identify high-risk patients with stage II colon cancer. Int J Biol Markers 32:e267–e273. doi:10.5301/jbm.5000255. 28478638

[33] Tatiana Z, Galina Z, Olga K, Ruslan Z, Marina P, Ana G, Maxim B, Anna K. 2017. Current and prospective protein biomarkers of lung cancer. Cancers 9:155. doi:10.3390/cancers9110155. PMC5704173 29137182

[34] Hengmin J, Liang Z, Baolong W. 2019. The value of combination analysis of tumor biomarkers for early differentiating diagnosis of lung cancer and pulmonary tuberculosis. Annals of Clinical and Laboratory Science 49:645–649. 31611208

[35] Robinson CJ, Young VB. 2010. Antibiotic administration alters the community structure of the gastrointestinal microbiota. Gut Microbes 1:279–284. doi:10.4161/gmic.1.4.12614. 20953272 PMC2954510

[36] Jakobsson HE, Jernberg C, Andersson AF, Sjölund-Karlsson M, Jansson JK, Engstrand L. 2010. Short-term antibiotic treatment has differing long-term impacts on the human throat and gut microbiome. PLoS One 5:e9836. doi:10.1371/journal.pone.0009836. 20352091 PMC2844414

[37] Dethlefsen L, Relman DA. 2011. Incomplete recovery and individualized responses of the human distal gut microbiota to repeated antibiotic perturbation. Proc Natl Acad Sci U S A 108:4554–4561. doi:10.1073/pnas.1000087107. 20847294 PMC3063582

[38] Hu Y, Yang Q, Liu B, Dong J, Sun L, Zhu Y, Su H, Yang J, Yang F, Chen X, Jin Q. 2019. Gut microbiota associated with pulmonary tuberculosis and dysbiosis caused by anti-tuberculosis drugs. J Infect 78:317–322. doi:10.1016/j.jinf.2018.08.006. 30107196

[39] Xu L, Surathu A, Raplee I, Chockalingam A, Stewart S, Walker L, Sacks L, Patel V, Li Z, Rouse R. 2020. The effect of antibiotics on the gut microbiome: a metagenomics analysis of microbial shift and gut antibiotic resistance in antibiotic treated mice. BMC Genomics 21. doi:10.1186/s12864-020-6665-2. PMC7106814 32228448

[40] Willmann M, Vehreschild MJGT, Biehl LM, Vogel W, Dörfel D, Hamprecht A, Seifert H, Autenrieth IB, Peter S. 2019. Distinct impact of antibiotics on the gut microbiome and resistome: a longitudinal multicenter cohort study. BMC Biol 17. doi:10.1186/s12915-019-0692-y. PMC6749691 31533707

[41] Mac Aogáin M, Lau KJX, Cai Z, Kumar Narayana J, Purbojati RW, Drautz-Moses DI, Gaultier NE, Jaggi TK, Tiew PY, Ong TH, Siyue Koh M, Lim Yick Hou A, Abisheganaden JA, Tsaneva-Atanasova K, Schuster SC, Chotirmall SH. 2020. Metagenomics reveals a core macrolide resistome related to microbiota in chronic respiratory disease. Am J Respir Crit Care Med 202:433–447. doi:10.1164/rccm.201911-2202OC. 32320621 PMC7397787

[42] Identificatio H. 2000. Diagnostic standards and classification of tuberculosis in adults and children. Am J Respir Crit Care Med 161:1376–1395. doi:10.1164/ajrccm.161.4.16141. 10764337

[43] Arango L, Brewin AW, Murray JF. 1973. The spectrum of tuberculosis as currently seen in a metropolitan hospital. American Rev Respir Dis 108:805–812. 10.1164/arrd.1973.108.4.805 4741875

[44] Chen S, Zhou Y, Chen Y, Gu J. 2018. fastp: an ultra-fast all-in-one FASTQ preprocessor. Bioinformatics 34:i884–i890. doi:10.1093/bioinformatics/bty560. 30423086 PMC6129281

[45] Andrews S. 2013. FastQC: a quality control tool for high throughput sequence data. Babraham Bioinformatics, Cambridge, UK. https://www.bioinformatics.babraham.ac.uk/projects/fastqc/.

[46] Langmead B, Salzberg SL. 2012. Fast gapped-read alignment with Bowtie 2. Nat Methods 9:357–359. doi:10.1038/nmeth.1923. 22388286 PMC3322381

[47] Wood DE, Salzberg SL. 2014. Kraken: ultrafast metagenomic sequence classification using exact alignments. Genome Biol 15:R46. doi:10.1186/gb-2014-15-3-r46. 24580807 PMC4053813

[48] Wood DE, Lu J, Langmead B. 2019. Improved metagenomic analysis with Kraken 2. Genome Biol 20. doi:10.1186/s13059-019-1891-0. PMC6883579 31779668

[49] Lu J, Breitwieser FP, Thielen P, Salzberg SL. 2017. Bracken: estimating species abundance in metagenomics data. Peerj Computer Science 3:e104. doi:10.7717/peerj-cs.104. PMC12016282 40271438

[50] Davis NM, Proctor DM, Holmes SP, Relman DA, Callahan BJ. 2018. Simple statistical identification and removal of contaminant sequences in marker-gene and metagenomics data. Microbiome 17:226. doi:10.1186/s40168-018-0605-2. PMC6298009 30558668

[51] R Core Team. 2013. R: a language and environment for statistical computing. R Foundation for Statistical Computing, Vienna, Austria. http://www.R-project.org/.

[52] Oksanen J, Blanchet FG, Kindt R, Legendre P, Minchin PR, O’hara RB, Simpson GL, Solymos P, Stevens MHH, Wagner H. 2013. Vegan: community ecology package. R Package Version 20-2 http://CRAN.Rproject.org/package=vegan.

[53] Wickham H. 2016. ggplot2: elegant graphics for data analysis. Springer, New York, NY. 10.1007/978-0-387-98141-3_9.

[54] Huson DH, Beier S, Flade I, Górska A, El-Hadidi M, Mitra S, Ruscheweyh H-J, Tappu R. 2016. MEGAN community edition-interactive exploration and analysis of large-scale microbiome sequencing data. PLoS Comput Biol 12:e1004957. doi:10.1371/journal.pcbi.1004957. 27327495 PMC4915700

[55] Segata N, Izard J, Waldron L, Gevers D, Miropolsky L, Garrett WS, Huttenhower C. 2011. Metagenomic biomarker discovery and explanation. Genome Biol 12:P47. doi:10.1186/gb-2011-12-s1-p47. PMC3218848 21702898

[56] Kaminski J, Gibson MK, Franzosa EA, Segata N, Dantas G, Huttenhower C. 2015. High-specificity targeted functional profiling in microbial communities with ShortBRED. PLoS Comput Biol 11:e1004557. doi:10.1371/journal.pcbi.1004557. 26682918 PMC4684307

[57] Kolde R, Kolde MR. 2015. Package ‘pheatmap’. R Package 1:790.

[58] Alcock BP, Raphenya AR, Lau TTY, Tsang KK, Bouchard M, Edalatmand A, Huynh W, Nguyen A-LV, Cheng AA, Liu S, Min SY, Miroshnichenko A, Tran H-K, Werfalli RE, Nasir JA, Oloni M, Speicher DJ, Florescu A, Singh B, Faltyn M, Hernandez-Koutoucheva A, Sharma AN, Bordeleau E, Pawlowski AC, Zubyk HL, Dooley D, Griffiths E, Maguire F, Winsor GL, Beiko RG, Brinkman FSL, Hsiao WWL, Domselaar GV, McArthur AG. 2020. CARD 2020: antibiotic resistome surveillance with the comprehensive antibiotic resistance database. Nucleic Acids Res 48:D517–D525. doi:10.1093/nar/gkz935. 31665441 PMC7145624

[59] Suzek BE, Wang Y, Huang H, McGarvey PB, Wu CH, Consortium U, The UniProt Consortium. 2015. UniRef clusters: a comprehensive and scalable alternative for improving sequence similarity searches. Bioinformatics 31:926–932. doi:10.1093/bioinformatics/btu739. 25398609 PMC4375400

[60] Csardi G, Nepusz T. 2006. The igraph software package for complex network research. InterJournal, Complex Systems 1695:1–9.

[61] Bastian M, Heymann S, Jacomy M. 2009. Gephi: an open source software for exploring and manipulating networks. Proc Int AAAI Conf Weblogs Soc Media 3:361–362.

