# Supplementary material for: Insights into the Unique Lung Microbiota Profile of Pulmonary Tuberculosis Patients Using Metagenomic Next-Generation Sequencing
Source: Microbiol Spectr. 2022 Feb 23;10(1):e01901-21. doi: 10.1128/spectrum.01901-21 (PMC8865484; doi:10.1128/spectrum.01901-21)
Supplement: SUPPLEMENTAL FILE 5 — Supplemental material. Download SPECTRUM01901-21_Supp_1_seq8.pdf, PDF file, 2.3 MB [file spectrum01901-21_supp_1_seq8.pdf]

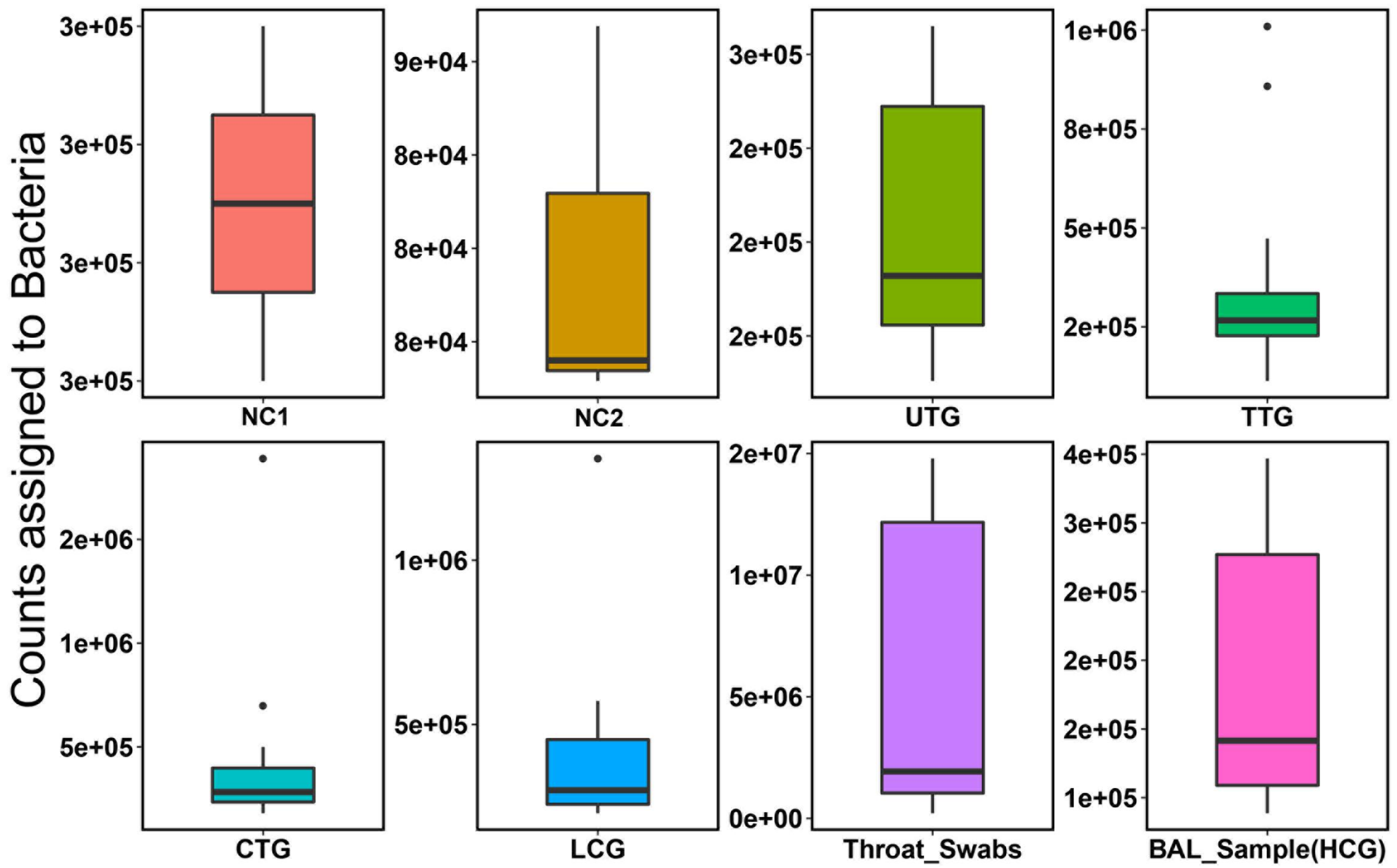

Figure S1: The average reads for each group matched to the bacterial domain. The vertical axis represents the number of average reads of each group matched to the bacterial domain. NC1: negative control 1, NC1: negative control 2, UTG: untreated TB group, TTG: treated TB group, CTG: cured TB group, LCG: lung cancer group, HCG: healthy control group.

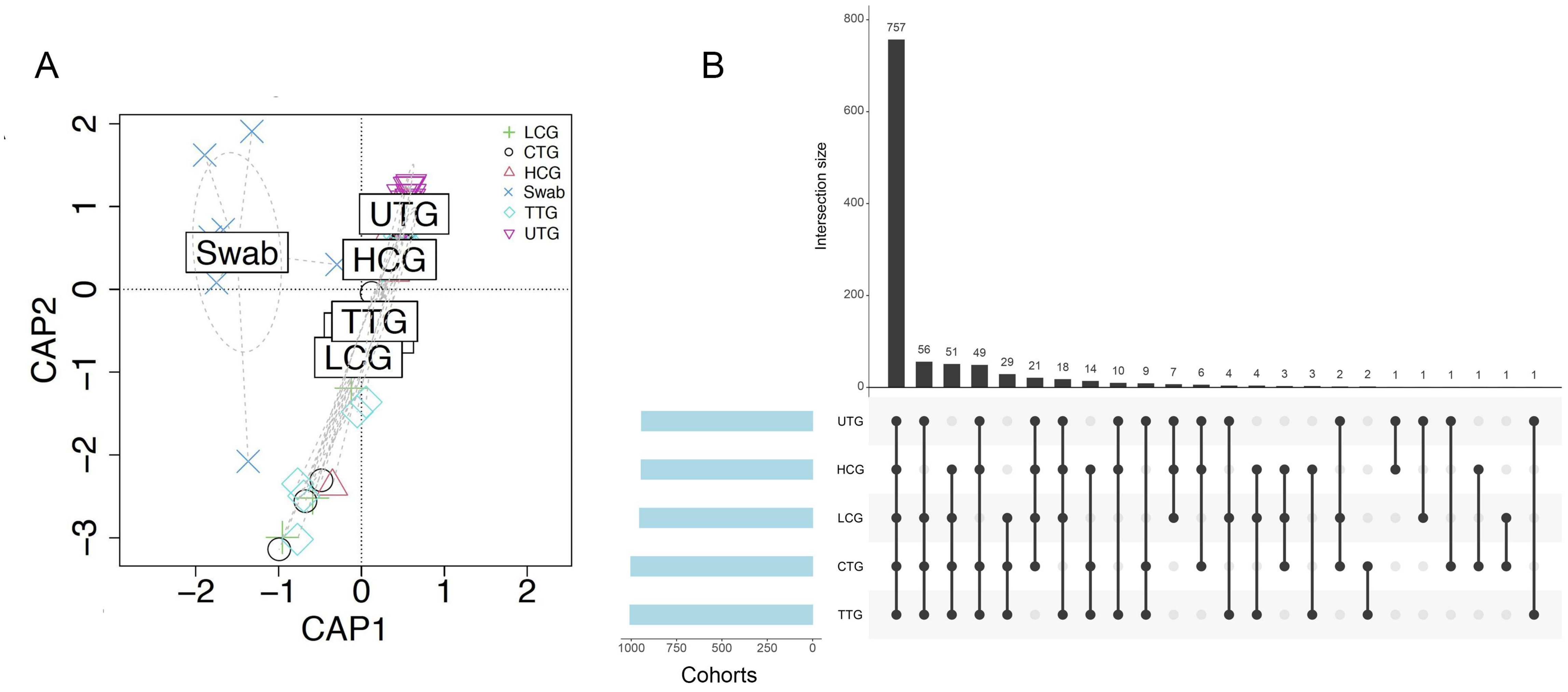

Figure S2: Overview of microbiota within all samples. A: Constrained analysis of principal coordinates (CAP) using bacterial genus relative abundances.The ellipses indicate throat swab samples. B: UpSet plots were used to determine the common genera within the indicated groups. The vertical bar charts represent the number of genera or species contained in each type of group. The vertical bar charts represent the number of genus or species included in each group. The dotted lines show the contained groups.

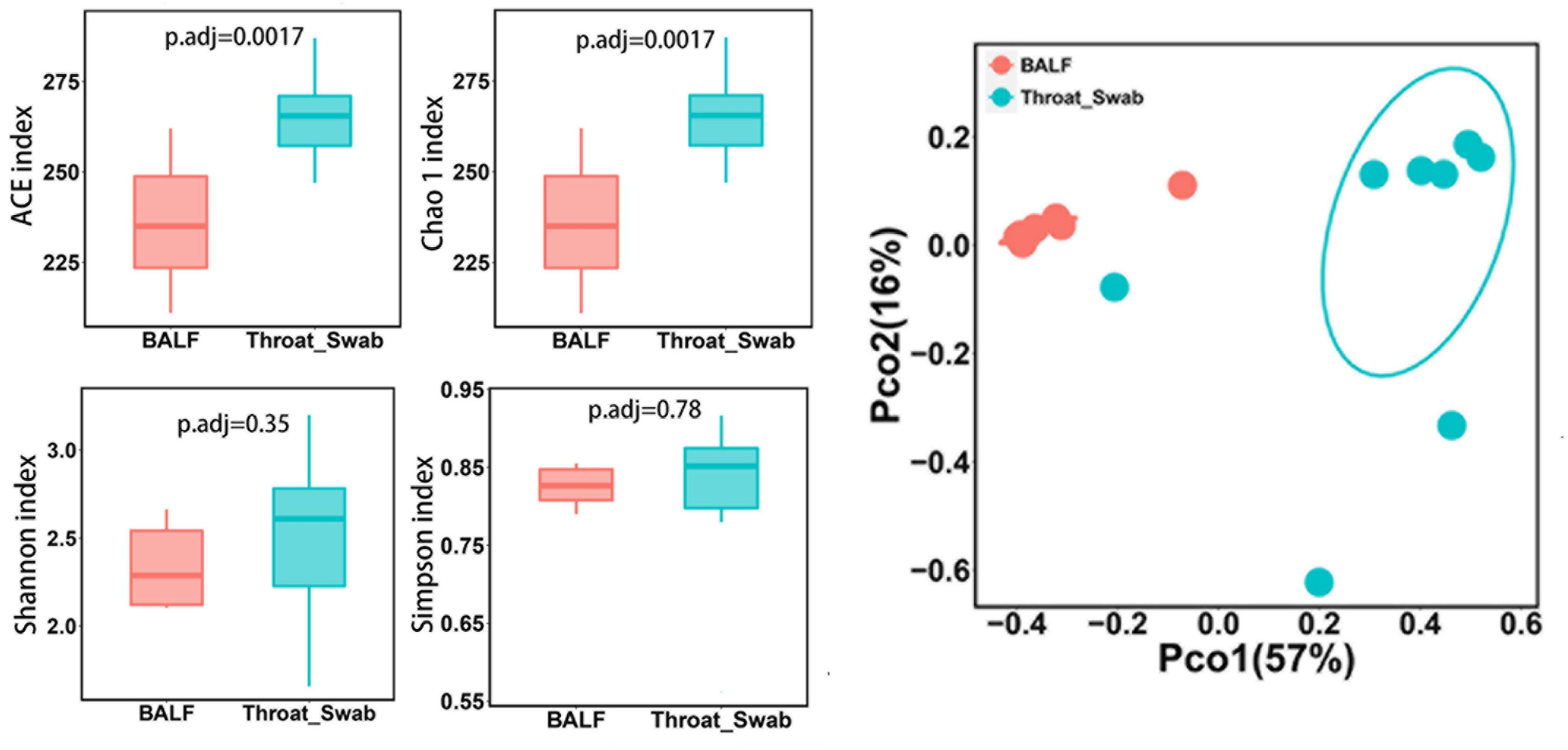

Figure S3: Microbiota profiles significantly differ between throat swabs and BALF samples from healthy individuals. Alpha diversity indicated by the ACE index, Chao1 index, Shannon index and Simpson index at the genus level. Adjusted p value are listed at the top of the bar charts. Beta diversity depicted by principle coordinates analysis (PCoA) plot based on Bray curtis dissimilarity at the genus level. Each dot represents one sample from each group.

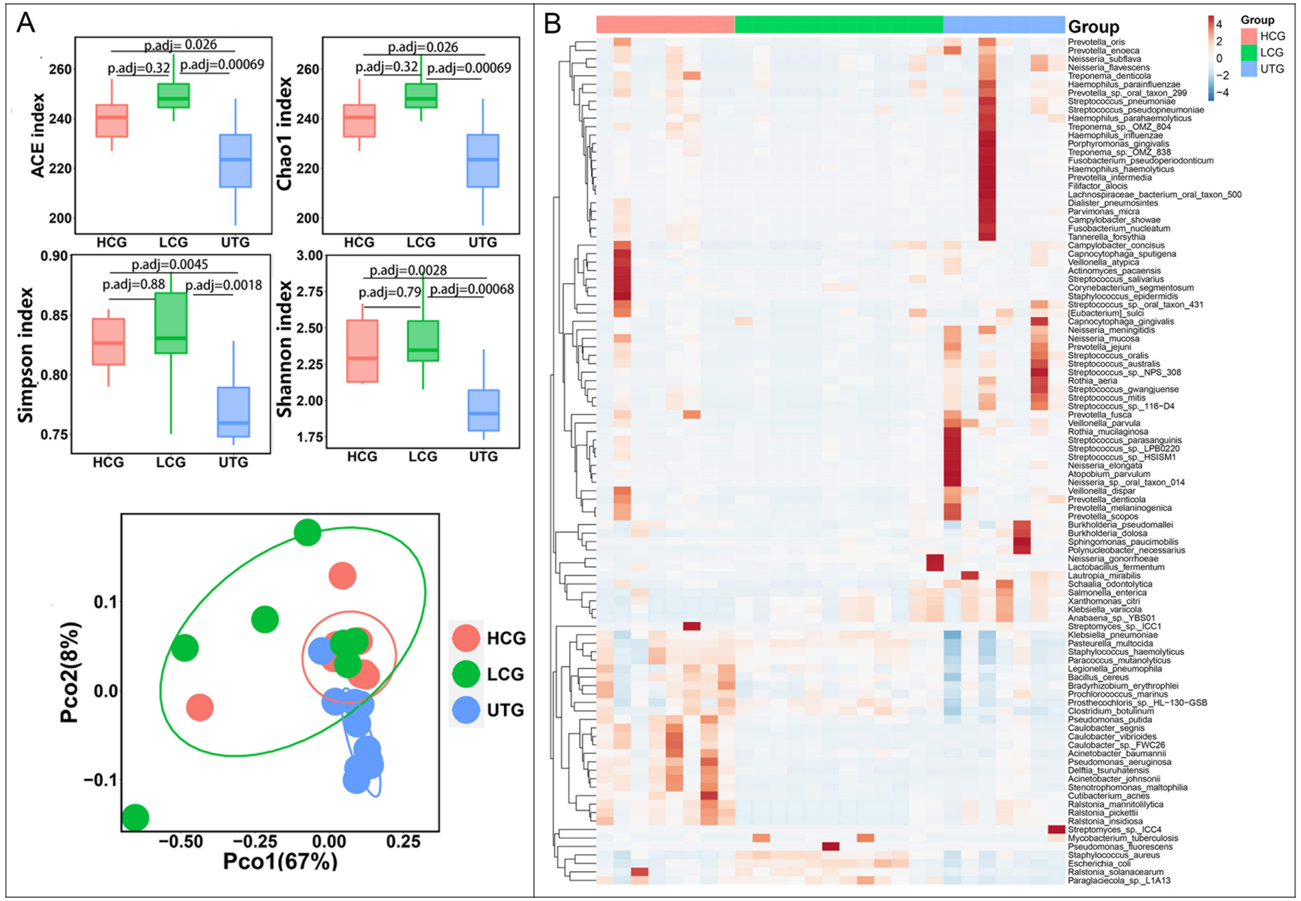

Figure S4: Comparison of lung microbiota among the UTG, HCG and LCG samples. A: Alpha diversity indicated by the ACE index, Chao1 index, Shannon index and Simpson index at the genus level. Adjusted p value were listed at the top of the bar charts. Beta diversity depicted by principle coordinates analysis (PCoA) plot based on Bray curtis dissimilarity at the genus level. Each dot represents one sample from each group. B: Heatmap of the top 100 species based on Euclidean distance. Bacterial species names are listed on the right side of the heatmap, and group names are indicated by color bars at the top of map. The dendrogram on the left of the map indicates the phylogenetic relationship between species.

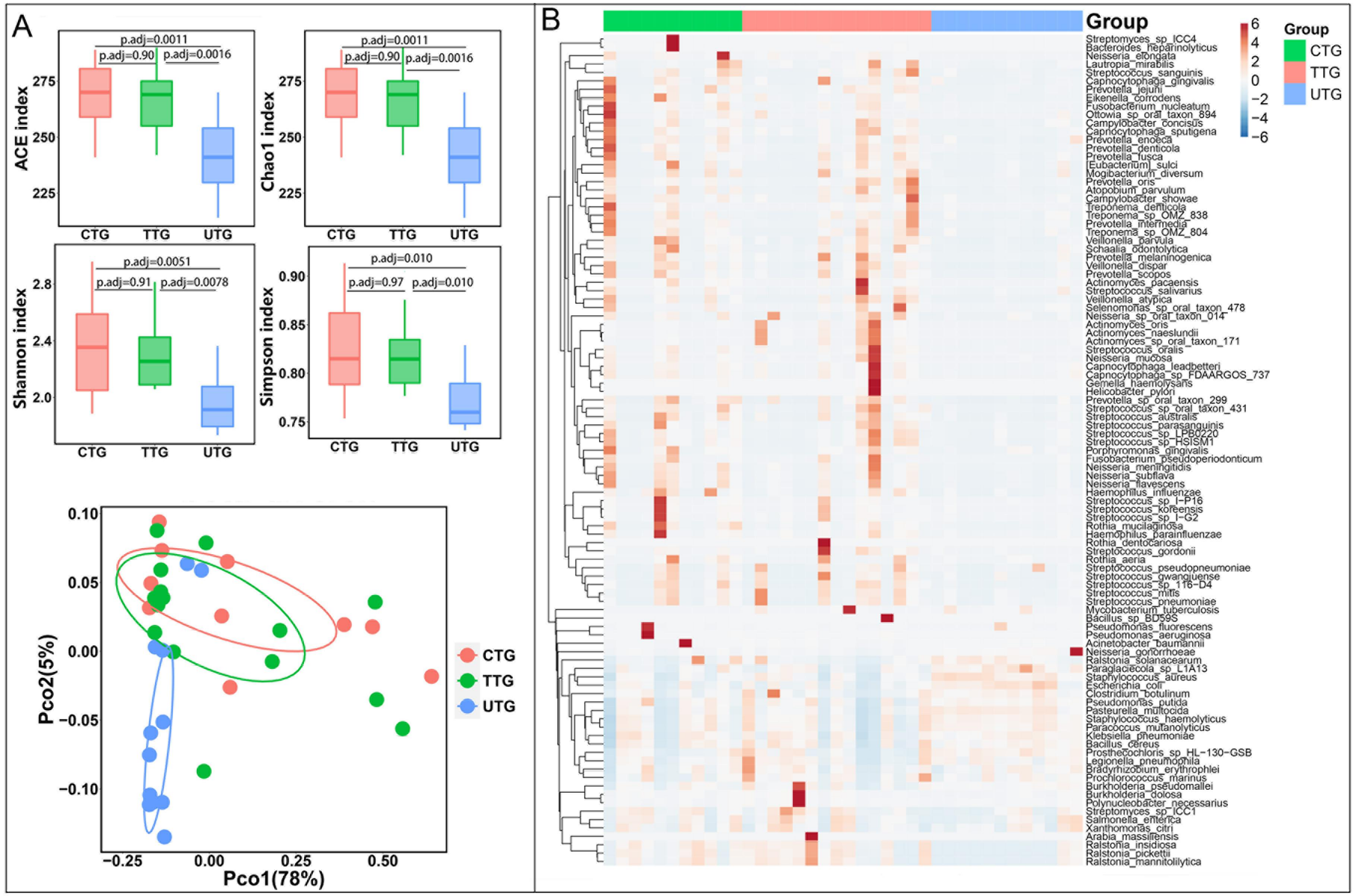

Figure S5: Anti-TB treatment significantly alters lung microbiota. A: Alpha diversity indicated by the ACE index, Chao1 index, Shannon index and Simpson index at the genus level. Adjusted p value are listed at the top of the bar charts. Beta diversity depicted by Principle Coordinates Analysis (PCoA) plot based on Bray curtis dissimilarity at genus level. Each dot represents one sample from each group; B: Heatmap of top 100 species based on euclidean distance. Bacterial species names are listed on the right side of the heatmap, and group names are indicated by color bars at the top of map. The dendrogram on the left of the map indicates the phylogenetic relationship between species.

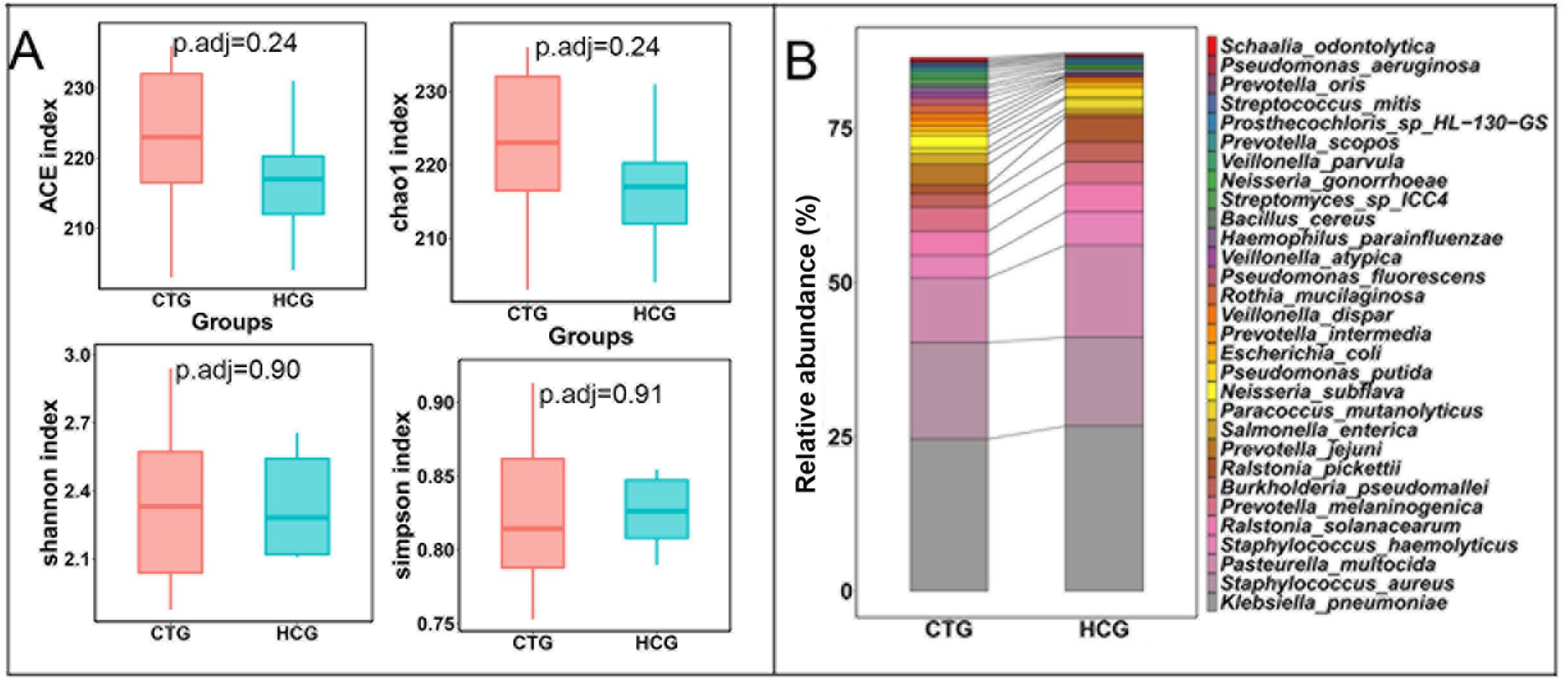

Figure S6: Comparison of lung microbiota between the CTG and HCG samples. A: Alpha diversity indicated by the ACE index, Chao1 index, Shannon index and Simpson index at the genus level. Adjusted p value are listed at the top of bar charts. B: Bar chart of the top 30 species in both groups.
